# Intranasal lidocaine for acute migraine: A protocol for the systematic review of randomized clinical trials

**DOI:** 10.1097/MD.0000000000015699

**Published:** 2019-05-17

**Authors:** Pei-Wen Chi, Kun-Yi Hsieh, Chien-Wei Tsai, Chin-Wang Hsu, Chyi-Huey Bai, Chiehfeng Chen, Yuan-Pin Hsu

**Affiliations:** a Emergency Department; b Department of Emergency; c Department of Public Health, School of Medicine, College of Medicine; d Cochrane Taiwan; e Division of Plastic Surgery, Department of Surgery; f Evidence-based Medicine Center, Wan Fang Hospital, Taipei Medical University, Taipei, Taiwan.

**Keywords:** headache, intranasal, lidocaine, migraine, transnasal, xylocaine

## Abstract

**Background:**

Intranasal lidocaine has been shown to be effective in treating patients with acute migraines; however, its efficacy is still controversial. The aim of our study is to assess the efficacy and safety of intranasal lidocaine compared with a placebo or an active comparator for the treatment of acute migraine.

**Methods:**

We will use PubMed, EMBASE, Cochrane library, and Scopus databases to search for articles from their inceptions to November 2018. We will only include randomized controlled studies. Data were independently will be extracted by 2 reviewers. Data analysis and synthesis will be analyzed by the Revman 5.3 software. We will conduct the study in accordance with the guideline of the Preferred Reporting Items for Systematic Review and Meta-analysis Protocols.

**Results:**

This review will evaluate the efficacy and safety of intranasal lidocaine for acute migraine. The primary outcome is pain intensity measured by visual analogue, numerical rating scale, or verbal rating scale. Secondary outcomes are success rate, requirement of rescue medicine, relapse, and adverse events.

**Conclusion:**

The findings of this systematic review will summarize the latest evidence of intranasal lidocaine for acute migraine. The results will provide implications for clinical practice and further research.

**Prospero registration number:** CRD42018116226

## 1 Introduction

Migraine is among the most common neurological disorders. Migraine headache is characterized by throbbing pain affecting one side of the head, and is aggravated by routine physical activity. The estimated global prevalence is around 10% to 15%; women are affected more than men with a ratio of 2 to 3 to 1.^[1,2]^ More than 1 million patients present to US emergency departments (ED) annually to obtain relief from acute migraine,^[3]^ but fewer than 25% of patients achieve the ultimate goal: complete and sustained relief after treatment of acute migraine in the ED.^[4]^

Medications commonly used as abortive treatment for acute migraine including triptan, antiemetics, ergotamine, and nonsteroidal anti-inflammatory drugs (NSAID). However, these drugs may have serious side effects such as development of a serotonin syndrome with triptan, tardive dyskinesia with antiemetics, vascular occlusion and rebound headaches for ergotamine, and gastrointestinal hemorrhage with NSAID. Therefore, there remains a need for an acute migraine intervention that can deliver rapid, complete, and sustained headache relief without causing side effects that prevent a patient from returning to work or usual activities.^[5]^

Intranasal lidocaine is viewed as a promising effective treatment for acute migraine.^[6–8]^ The strength of intranasal lidocaine administration is rapid effectiveness, lack of a need for an injection site, and rare adverse reactions. The proposed mechanism for intranasal lidocaine to relieve migraine is that the blockade of sphenopalatine ganglion decreases signals to intracranial nociceptors innervating migraine pain.^[6–9]^ However, its efficacy is still controversial.^[7–10]^

The hypothesis of our study is that among patients who presented with acute migraine, intranasal lidocaine would provide more pain intensity reduction, greater rates of short-term, and sustained headache relief when compared with a placebo. The administration of a comedication may be a confounding factor. By using a systematic search, we have included randomized controlled trials (RCTs) that investigate the efficacy and safety of intranasal lidocaine versus placebo for acute migraine. We will synthesize the results and explore a potential effect modifier through a subgroup analysis.

## 2 Methods

We will follow the Preferred Reporting Items for Systematic Review and Meta-Analysis Protocols (PRISMA-P).^[11]^ We registered on PROSPERO (https://www.crd.york.ac.uk/prospero, PROSPERO ID: CRD42018116226).

### 2.1 Ethics

Ethical approval or patient consent was not required as the present study was a review of previously published articles.

### 2.2 Criteria for included studies

#### 2.2.1 Study type

We will only include RCTs that evaluated intranasal lidocaine for acute migraine. Cohort studies, case series, case reports will be excluded

#### 2.2.2 Participants

The target population should be acute migraineur. Migraine type (e.g., migraine with aura, migraine without aura), duration of migraine, or frequency of attack will not be restricted. We will also include the study if the target population of the study was patients with primary headache but a subset of the migraine has been analyzed.

#### 2.2.3 Interventions and comparisons

The patients in the intervention group have intranasal lidocaine administered through any applicator. The patients in the control group can be treated with placebo or active comparator.

#### 2.2.4 Outcome

The primary outcome of interest will be pain intensity that was measured by visual analogue, numerical rating scale, or verbal rating scale. Secondary outcomes will be success rate, requirement of rescue medicine, relapse, and adverse events.

### 2.3 Search strategy and study selection

PubMed, EMBASE, Cochrane library, and Scopus databases will be searched from their inceptions to November 2018. We will explore eligible studies with the following search terms: lidocaine, xylocaine, intranasal, transnasal, headache, and migraine. We will not use filter or restrict language. We will manually check references of identified studies. Finally, we will search ClinicalTrials.gov registry (http://clinicaltrials.gov/) for any associated ongoing or unpublished studies. We have provided the sample of detailed search strategy for PubMed database in Table 1. The identical search strategies for other databases will be built and applied.

**Table 1:**
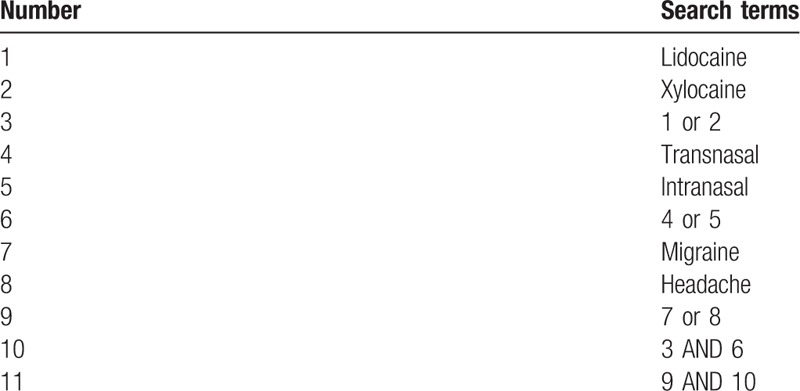
Search strategy applied in PubMed database.

### 2.4 Data collection

#### 2.4.1 Study selection

**Figure 1:**
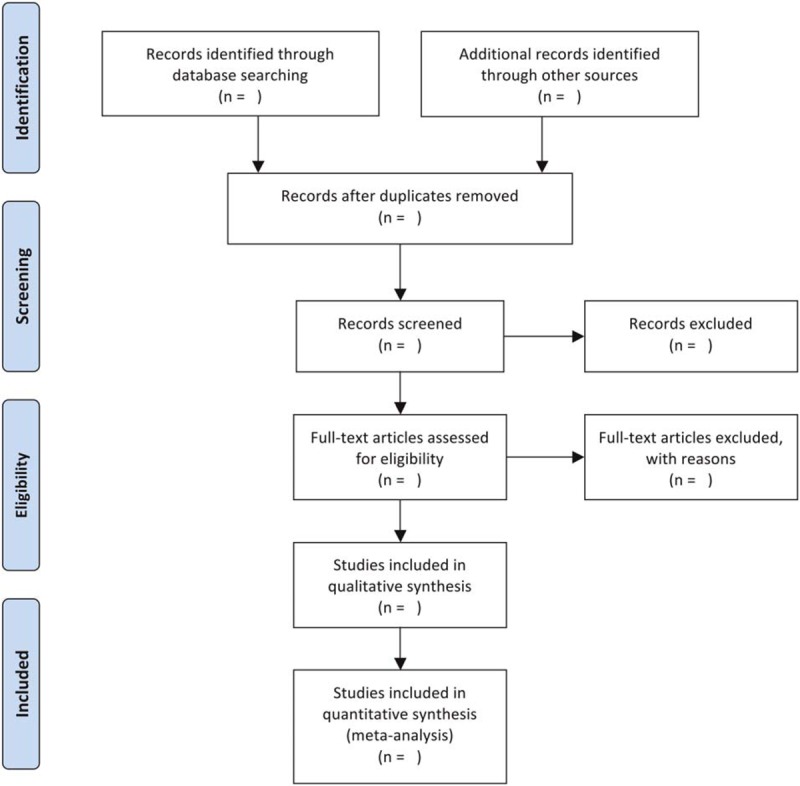
Flowchart of study selection.

Two reviewers will independently export the citation from the databases, check duplicated references, screen the titles and abstracts, as well as read full-texts if studies meet the predefined inclusion and exclusion criteria. Any divergences regarding the study selection between the 2 reviewers will be consulted with a third reviewer. Figure 1 details the procedures of study selection.

#### 2.4.2 Data extraction

Two reviewers will independently extract relevant data from each eligible RCT and place it into an electronic data-extraction sheet. The major information that will be extracted include:

(1) first author, publication year, country
(2) characteristics of the study population
(3) number of participants
(4) regimens of each comparison
(5) eligible outcome data
(6) time of follow-up

If any insufficient or missing data are identified, we will contact the primary authors for elaboration. If those data cannot be acquired, we will analyze the available data, and identify this issue in the discussion.

#### 2.4.3 Risk of bias assessment

Two reviewers will independently rate the quality of the included studies using the Revised Cochrane risk of bias tool (RoB 2.0) for RCTs.^[12]^ The tool includes 6 domains (bias arising from the randomization process, bias due to deviations from the intended intervention, bias due to missing outcome data, bias in measurements of outcomes, bias in selection of the reported result, and other biases).^[12]^ Each item is classified as high, unclear, and low risk of bias.^[12]^ A third and senior author will be involved to solve any of the disagreements.

### 2.5 Statistical analysis

#### 2.5.1 Measures of the treatment effect

We will perform the statistical analysis with a random effect model by using RevMan 5.3 (Copenhagen, Denmark). Continuous data will be synthesized and shown as mean difference (MD) or standardized mean difference (SMD) with 95% confidence intervals (CIs). The dichotomous data will be synthesized and presented as risk ratio (RR) with 95% CIs. A 2-sided *P* value of <.05 was considered statistically significant.

#### 2.5.2 Assessment of heterogeneity

The heterogeneity of the data will be assessed by *Q*-test and *I*^2^ statistic. We will regard the heterogeneity as low when *I*^2^ <50%, as moderate when *I*^2^ is 50% to 75%, and high when *I*^2^ >75%. If substantial heterogeneity is identified, we will explore possible causes base on the use of comedication.

#### 2.5.3 Assessment of reporting biases

We also plan to conduct funnel plot and Egg regression to detect the reporting bias if >10 eligible studies are included.^[13]^

## 3 Discussion

To the best of our knowledge, this is the first systematic review and meta-analysis protocol to evaluate intranasal lidocaine for the treatment of patient with acute migraine. In this systematic review, we will search as many comprehensive data sources as possible without any restrictions. All potential RCTs that evaluate intranasal lidocaine for acute migraine will be fully considered. The result will provide a rigorous summary evidence to determine whether or not intranasal lidocaine is an effect abortive treatment for acute migraine. The findings of this study may also bring helpful evidence for clinicians.

## Author contributions

Pei-Wen Chi, Kun-Yi Hsieh, and Yuan Pin Hsu had full access to all of the data in the study and take responsibility for the integrity of the data and the accuracy of the data analysis.

**Acquisition, analysis, or interpretation of data:** Pei-Wen Chi, Kun-Yi Hsieh, Chin-Wang Hsu, Chyi-Huey Bai, and Chiehfeng Chen.

**Statistical analysis**: Chiehfeng Chen, Chyi-Huey Bai, and Yuan Pin Hsu.

**Study supervision**: Yuan Pin Hsu.

**Conceptualization:** Yuan-Pin Hsu.

**Data curation:** Yuan-Pin Hsu.

**Formal analysis:** Kun-Yi Hsieh, Pei-Wen Chi, Chien-Wei Tsai, Chin-Wang Hsu, Chyi-Huey Bai, Chiehfeng Chen, Yuan-Pin Hsu.

**Investigation:** Kun-Yi Hsieh, Pei-Wen Chi, Chin-Wang Hsu, Chyi-Huey Bai, Chiehfeng Chen, Yuan-Pin Hsu.

**Methodology:** Kun-Yi Hsieh, Pei-Wen Chi, Chien-Wei Tsai, Chin-Wang Hsu, Chyi-Huey Bai, Chiehfeng Chen, Yuan-Pin Hsu.

**Project administration:** Kun-Yi Hsieh, Pei-Wen Chi, Chien-Wei Tsai, Chin-Wang Hsu, Chyi-Huey Bai, Chiehfeng Chen, Yuan-Pin Hsu.

**Resources:** Chien-Wei Tsai, Chiehfeng Chen, Yuan-Pin Hsu.

**Software:** Yuan-Pin Hsu.

**Supervision:** Yuan-Pin Hsu.

**Validation:** Yuan-Pin Hsu.

**Visualization:** Chin-Wang Hsu, Chyi-Huey Bai, Chiehfeng Chen, Yuan-Pin Hsu.

**Writing – original draft:** Kun-Yi Hsieh, Pei-Wen Chi.

**Writing – review & editing:** Yuan-Pin Hsu.

Yuan-Pin Hsu orcid: 0000-0001-8895-9133.

Pei-Wen Chi orcid: 0000-0001-7258-4541.

Kun-Yi Hsieh orcid: 0000-0003-4808-746X.

Chien-Wei Tsai orcid: 0000-0003-2842-5361.

Chin-Wang Hsu orcid: 0000-0003-4973-1050.

Chyi-Huey Bai orcid: 0000-0002-4658-1088.

Chiehfeng Chen orcid: 0000-0002-1595-6553.
